# Enrichment of type I interferon signaling in colonic group 2 innate lymphoid cells in experimental colitis

**DOI:** 10.3389/fimmu.2022.982827

**Published:** 2022-10-04

**Authors:** Emi Irie, Rino Ishihara, Ichiro Mizushima, Shunya Hatai, Yuya Hagihara, Yoshiaki Takada, Junya Tsunoda, Kentaro Iwata, Yuta Matsubara, Yusuke Yoshimatsu, Hiroki Kiyohara, Nobuhito Taniki, Tomohisa Sujino, Kaoru Takabayashi, Naoki Hosoe, Haruhiko Ogata, Toshiaki Teratani, Nobuhiro Nakamoto, Yohei Mikami, Takanori Kanai

**Affiliations:** ^1^ Division of Gastroenterology and Hepatology, Department of Internal Medicine, School of Medicine, Keio University, Tokyo, Japan; ^2^ Laboratory for Innate Immune Systems, Department of Microbiology and Immunology, Graduate School of Medicine, Osaka University, Osaka, Japan; ^3^ Department of Surgery, School of Medicine, Keio University, Tokyo, Japan; ^4^ Center for Diagnostic and Therapeutic Endoscopy, School of Medicine, Keio University, Tokyo, Japan; ^5^ AMED-CREST, Japan Agency for Medical Research and Development, Tokyo, Japan

**Keywords:** ILC2 - group 2 innate lymphoid cell, type I interferon, ifnar, colitis, ibd

## Abstract

Group 2 innate lymphoid cells (ILC2s) serve as frontline defenses against parasites. However, excluding helminth infections, it is poorly understood how ILC2s function in intestinal inflammation, including inflammatory bowel disease.** **Here, we analyzed the global gene expression of ILC2s in healthy and colitic conditions and revealed that type I interferon (T1IFN)-stimulated genes were up-regulated in ILC2s in dextran sodium sulfate (DSS)-induced colitis. The enhancement of T1IFN signaling in ILC2s in DSS-induced colitis was correlated with the downregulation of cytokine production by ILC2s, such as interleukin-5. Blocking T1IFN signaling during colitis resulted in exaggeration of colitis in both wild-type and *Rag2*-deficient mice. The exacerbation of colitis induced by neutralization of T1IFN signaling was accompanied by reduction of amphiregulin (AREG) in ILC2s and was partially rescued by exogenous AREG treatment. Collectively, these findings show the potential roles of T1IFN in ILC2s that contribute to colitis manifestation.

## Introduction

Innate lymphoid cells (ILCs) are a population of lymphocytes that lack an antigen-specific receptors. ILCs were initially classified into three subsets, group 1, 2, and 3 ILCs, based on the expression pattern of signature cytokines and lineage-determining transcription factors (1) and are now recognized as five subsets, natural killer cells, ILC1, ILC2, ILC3, and lymphoid tissue inducer cells, based on their development and function (2). ILCs are enriched at mucosal sites, including the gut, and play critical roles, particularly ILC3s, in the early immune response, tissue protection, and maintenance of intestinal integrity (2, 3). In line with its importance in the gut, the potential roles of ILCs in the context of intestinal inflammation in inflammatory bowel disease (IBD) and its animal models have been gradually recognized (4, 5). ILC3s are one of the most extensively studied classes of ILCs in the context of intestinal inflammation and are known to induce interleukin (IL)-22, which promotes epithelial regeneration and production of antimicrobial peptides (6). ILC2s provide an early source of Th2 cytokines, serve as a frontline defense against parasites such as helminths, and are important part of tumor immunity (7–9). Therefore, it is highly likely that ILC2s contribute to the manifestation of intestinal inflammation (4). However, the precise roles of ILC2s in intestinal inflammation and IBD are poorly understood.

Herein, we focused on the roles of colonic ILC2s and analyzed its transcriptome in healthy and diseased conditions to identify molecular networks chiefly regulated during murine experimental colitis. We demonstrated that ILC2s are the most abundant cells found in the small and large intestine than in the spleen and lymph nodes. In dextran sodium sulfate (DSS)-induced colitis, IL-5, the hallmark cytokine of ILC2s, was significantly reduced, while the type I interferon (T1IFN) signature was enriched. To investigate the role of T1IFN signaling in ILC2s, we assessed mice treated with anti-T1IFN receptor and observed reduced amphiregulin (AREG) production in ILC2s and exaggeration of colitis in wild-type (WT) and *Rag2*-deficient mice, which was rescued by supplementation with AREG. Our research highlights the potential protective mechanism of ILC2s through T1IFN signaling.

## Methods

### Mice

C57BL/6 WT mice were purchased from CLEA Japan (Tokyo, Japan). They were maintained in the germ-free (GF) facility of the Keio University School of Medicine. Sex (female)-matched mice aged 6 to 8 weeks were used in the experiments. *Rag2*-deficient mice were obtained from the Taconic Laboratory. All experiments were approved by the regional animal study committees (Keio University) and performed according to institutional guidelines and home office regulations.

### DSS-induced colitis model

Colitis was induced in mice using 2.5% DSS solution in drinking water for 7 days. The mice were weighed daily and visually inspected for diarrhea and rectal bleeding. The disease activity index (DAI) was assessed in each mouse group (maximum total score, 12) (10).

### Isolation of colonic lamina propria mononuclear cells in mice

Lamina propria mononuclear cells were isolated as described in the previous studies (11). The dissected colon mucosa was cut into 5mM pieces. Tissue was incubated with Ca2^+^ and Mg2^+^-free HBSS containing 1 mM dithiothreitol (DTT) and 5 μM Ethylenediaminetetraacetic acid (EDTA) at 37°C for 30 min, followed by further digestion with collagenase and DNase for 30 min. The cells were separated using a Percoll density gradient. The numbers of live cells was determined using the Countess II (Thermo Fisher Scientific).

### Flow cytometry and cell sorting

After blocking with anti-mouse CD16/CD32 antibody for 20 min, the cells were incubated with the specific fluorescence-labelled monoclonal antibodies at 4°C for 30 min, followed by permeabilization with Foxp3/Transcription Factor Fixation/Permeabilization Concentrate and Diluent (eBioscience) and intracellular staining. The following monoclonal antibodies were used for the fluorescence-activated cell sorting (FACS) analysis: anti-mouse CD45.2, CD45, CD3e, CD5, CD19, NK1.1, B220, KLRG1, GATA3, IL-5, IL-13, IL-17A, IL-22, IFN-γ, amphiregulin, FOXP3, T-bet, and RORγt. Dead cells were excluded using the Fixable Viability Dye eFluor. Events were acquired with FACS Canto II or LSRFortessa (BD Biosciences) and analyzed using FlowJo software (BD Biosciences). Colonic ILC2s (CD127^+^NK1.1^−^CD3^−^CD5^−^CD19^−^B220^−^KLRG^+^ cells) were stained after using EasySep Mouse ILC2 Enrichment Kit (STEMCELL Technologies) to remove other cells and sorted using BD FACSAria II (BD Biosciences). See **Supplementary Table 1** for information on the antibodies.

### RNA sequencing

RNA sequencing (RNA-seq) was performed and analyzed as described in the previous studies (12). Total RNA was prepared from approximately 10,000-50,000 cells by using the TRIzol reagent and was subsequently processed to generate an mRNA-seq library using the NEBNext Poly(A) mRNA Magnetic Isolation Module (NEB, E7490S), NEBNext Ultra II Directional RNA Library Prep with Sample Purification Beads (NEB, E7765S), and NEBNext Multiplex Oligos for Illumina (Index Primers Set 1 and 2) (NEB, E7335S, and E7500S), according to the manufacturer’s protocol. The libraries were sequenced for 150-bp paired-end read using Illumina HiSeq X Ten. To quantify transcript abundance, we pseudo-aligned RNA-seq reads to ENSEMBL transcripts (release 95 GRCm38), using Kallisto (v.0.44.0, options: -b 100) (13). Differentially expressed transcripts were identified using the sleuth R package. Transcript abundances were output by Kallisto in transcripts per million (TPM). Differentially expressed genes (DEGs) were calculated as genes with a sleuth q-value of < 0.05, fold change > 2, and expression > 10 TPM in at least one condition. Enrichment analyses of DEGs were performed using Metascape (http://metascape.org). Pathway analysis of DEGs were performed using Ingenuity pathway analysis (IPA) (Qiagen, Denmark).

### Single-cell RNA sequencing

Colonic CD45^+^EpCAM^−^ live cells were sorted from the pooled colonic mononuclear cells and loaded into a chromium controller (10X Genomics). RNA-seq libraries were then prepared using the Chromium Single Cell 3′ Reagent Kit v2 according to the manufacturer’s instructions (10X Genomics, CA, USA). The generated scRNA-seq libraries were sequenced using 150 cycles (paired-end reads) with a HiSeq X (Illumina, CA, USA).

### *In vitro* culture

For *in vitro* experiments, 1.0×10^6^ cells per well were cultured in a 24-well flat bottom plate in complete medium. Depending on the experiment, different combinations of 100 ng/mL IL-25 (R&D), 100 ng/mL IL-33 (Pepro Tech), or Phorbol-12-myristate-13-acetate (PMA) plus ionomycin were added and cultured for 4 h in a humidified incubator at 37°C. To stimulate *lamina propria* mononuclear cells (LPMC) for 24 h *in vitro*, 2.0×10^5^ cells were cultured with 10 ng/ml IFN-β (PBL, NJ, USA) and 100ng/ml IL-33 or PMA plus ionomycin in complete medium in a 96-well plate.

### *In vivo* cytokine treatment

To treat DSS-induced intestinal inflammation, mice were injected intraperitoneally (i.p.) with 1 mg of either IFN-alpha/beta receptor (IFNAR) blocking antibody (Ab), MAR1-5A3 (BioXCell, NH, USA),an isotype control Ab, MOPC-21 (BioXCell, NH, USA) or PBS on day 0 (14). Recombinant murine AREG (400 μg/kg; carrier-free, R &D) was administered i.p. on days 2, 4, and 6.

### Histological assessment of intestinal inflammation

Colon samples were fixed in buffered 10% formalin and embedded in paraffin. Paraffin-embedded colon sections were stained with hematoxylin and eosin and then examined. The histological activity score (maximum total score, 40) was assessed as the sum of three parameters: extent, inflammation, and crypt damage related to the percentage of involvement of the mucosal surface in each slide (15).

### Statistics

Prism (GraphPad Software) was used for statistical analyses. Data were tested using unpaired two-tailed Student’s *t*-test or one-way ANOVA, as indicated. Data are presented as the mean ± SEM. Statistical significance was set at *P* < 0.05.

## Result

### ILC2s are the most frequent population among the colon ILCs

**Figure 1:**
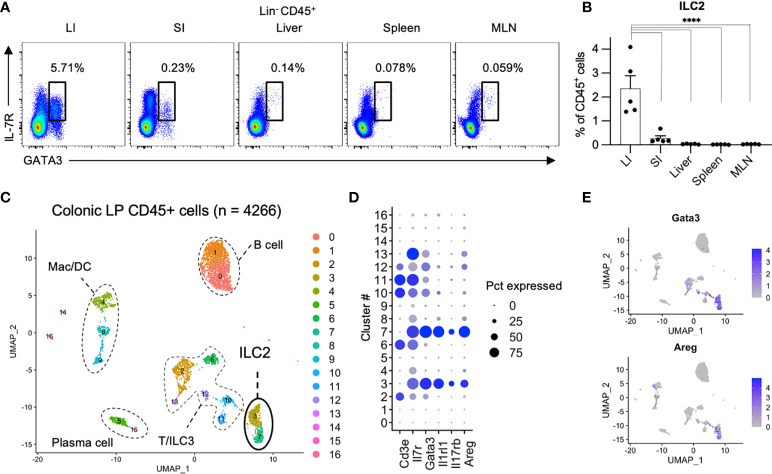
Comparison of ILC2s across digestive organs **(A, B)** Representative flow cytometry plots **(A)** and frequencies among CD45^+^ lymphocytes **(B)** of Lin^−^CD127^+^GATA3^+^ ILC2s in the colon (LI), small intestine (SI), liver, spleen, and mesenteric lymph nodes (MLN) of SPF (specific pathogen free) mice. n=5 for each tissue. Data are representative of two independent experiments (mean and SEM in B). **(C–E)** ScRNA-seq analysis of colonic CD45^+^ cells. **(C)** Uniform manifold approximation and projection (UMAP) of CD45^+^ cells (n = 4266) derived from pooled 5 SPF mice, showing the formation of 16 main clusters represented by different colors. The functional description of each cluster is determined by the gene expression characteristics of each. **(D)** Dot plot visualizing the expression of representative genes of ILC2s. The color represents the average expression level, and the circle size represents the proportion of cells expressing each gene. **(E)** Expression levels of the specified marker genes on the UMAP plots. ****P < 0.0001.

We first investigated the distribution of ILCs in the digestive and immune system organs of the peritoneal cavity, including the colon, small intestine, liver, spleen, and mesenteric lymph nodes (MLN). ILCs were gated on CD45^+^ and CD127 (IL-7R)^+^ cells after removing lineage markers (Lin; CD3, CD5, CD19, and B220) with lymphoid morphology (1, 7, 16). In line with the roles of frontline defenses at the mucosal surface, ILCs are mainly populated in the colon and small intestine (**Supplementary Figure 1A**). Among the total ILCs, the proportion of ILC2s as transcription factor GATA3^+^ cells among Lin^−^ CD45^+^ CD127^+^ cells (**Figure 1A**) (1) was significantly higher in the colon than in the small intestine, spleen, and MLN (**Figure 1B**). This finding is consistent with a previous study showing the predominance of ILC2s among total ILC subsets in rats (16). To confirm the profile of immune cells in the colonic *lamina propria*, we generated an scRNA-seq library of 4266 immune cells purified as CD45^+^ EpCAM^−^ live cells from the colonic *lamina propria* of healthy mice. The immune cells were unbiasedly clustered into 16 subsets, including T cells (cluster (Cl) 2, 6, 10, 11, and 12; *Cd3e*), B cells (Cl 0 and 1;*Cd19*), plasma cells (Cl 5 and 15; *Igha*), macrophages and dendritic cells (Cl 4, 8, and 9; *Csf1r*, *Itgam*), and ILCs (Cl 3, 7, and 13; *Il7r*) (**Figure 1C** and **Supplementary Figure 1B**). Substantial proportion of ILC2s were identified as *Cd3e* ^−^ *Il7r* ^+^ *Gata3* ^+^ (Cl 3 and 7) and expressed genes encoding signature cytokines and growth factors, such as *Il5*, *Il13*, *Calca*, and *Areg*, which is consistent with previous reports (17–20) (**Figures 1D, E**, **Supplementary Figure 1B**). These results highlight that ILC2s are the dominant ILC subset in colonic tissues.

### Type I interferon-stimulated genes were up-regulated in ILC2s in DSS-induced colitis

**Figure 2:**
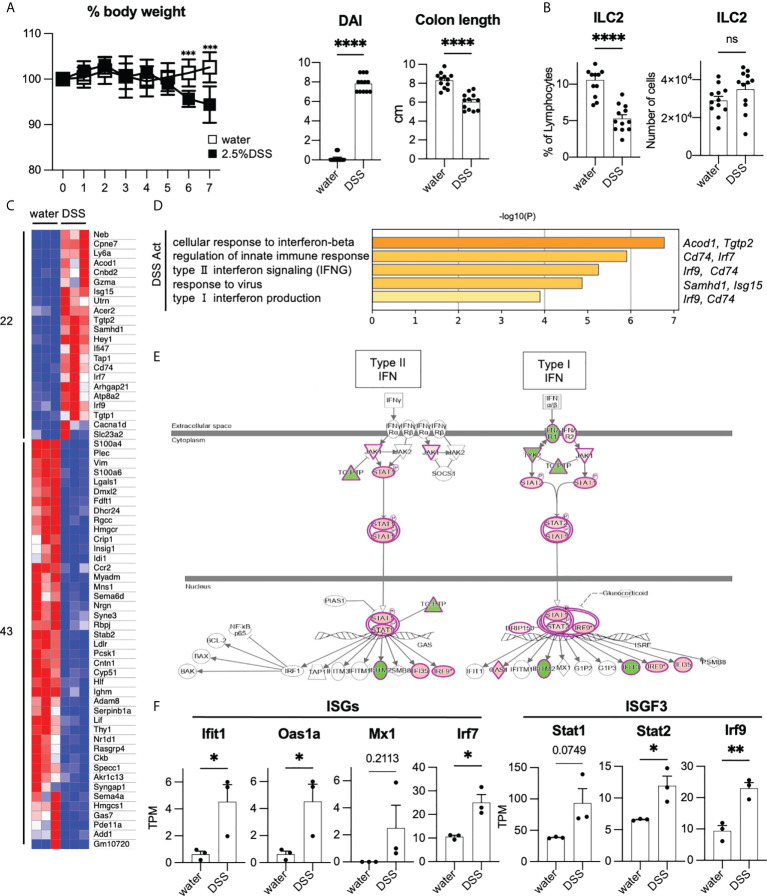
Enhancement of T1IFN signaling in ILC2s in DSS-induced colitis **(A, B)** WT mice were administered DSS for 7 days. Graphs show pooled data from three independent experiments (n=12 for each group). **(A)** Change in % body weight relative to initial body weight of control mice (drinking water) and DSS-treated mice in two representative experiments (n=8 for each group). Disease activity index (DAI) score on day 7 and colon length of mice of each group (n=12 for each group). **(B)** % lymphocyte and absolute number of colonic ILC2s in each group (n=12 for each group). **(C)** Heatmap showing relative gene expression of highly expressed differentially expressed (DE) genes (max TPM>10, log2FC>1, log2FC<-1) and the number of upregulated (22 genes) and downregulated genes (43 genes) in DSS treated (n=3) compared to that of control mice (n=3). **(D)** Gene Ontology (GO) analysis of the DSS-activated genes defined in panel C including names of representative genes. **(E)** Ingenuity pathway analysis (IPA) of interferon signaling pathway of genes differentially expressed in DSS-induced colitis. **(F)** Absolute gene expression (TPM) of Type I IFN related genes. Data are mean and SEM in **(A, B, F)** ns, not significant; *P < 0.05, **P < 0.01, ***P < 0.001, ****P < 0.0001.

To investigate the phenotype of ILC2s in colitis, we fed mice with 2.5% DSS to induce acute colonic inflammation. DSS-induced colitis is a commonly used mouse model for investigating the pathology of IBD (21). DSS-treated mice showed significantly more sever body weight loss, shorter colon length, and higher DAI than that of the control mice (**Figure 2A**). Post 7 days of DSS treatment, the frequency of colonic ILC2s among lymphocytes decreased; however, the numbers of ILC2s were comparable in DSS-induced colitis and control mice (**Figure 2B**). We next assessed the cytokine production in ILC2s and observed that production of IL-5, but not IL-13, was significantly reduced in DSS-induced colitis when stimulated by PMA plus ionomycin (**Supplementary Figures 2A, B**). We also confirmed that colonic ILC2s obtained from DSS-induced colitis mice showed significantly reduced IL-5 production only when stimulated with IL-25 and IL-33 alone or in combination (**Supplementary Figure 2C**).

Given that the phenotype of colonic ILC2s is altered in DSS-induced colitis, we assessed the global gene expression of ILC2s under healthy and colitic conditions (sorting strategy is shown in **Supplementary Figure 2D**). First, we analyzed the signature genes of ILC2s (22). As expected, substantial levels of ILC2s’ signature genes, such as *Gata3*, *Klrg1*, *Rora*, *Il1rl1*, *Areg*, *Il5*, and *Il13*, were detected in ILC2s in both groups, while signature genes for ILC1 and ILC3 were at marginal levels (**Supplementary Figures 2E, F**). To elucidate the phenotypic changes of ILC2s during colitis, we used DEGs as shown in the heatmap (**Figure 2C**) and performed gene ontology (GO) analysis (**Figure 2D**). GO analysis indicated that genes related to T1IFN and IFN-γ signaling or response to viruses were significantly enriched in ILC2s in DSS-induced colitis. Consistently, pathway analysis also showed the upregulation of genes related to T1IFN signaling, such as interferon-stimulated genes (ISGs) and IFN-stimulated gene factor3 (ISGF3) in ILC2s in DSS-induced colitis (**Figures 2E, F**). These data suggest that T1IFN signaling is enhanced in colonic ILC2s during colitis.

### Neutralization of T1IFN signaling reduced amphiregulin in ILC2s in DSS-induced colitis

**Figure 3:**
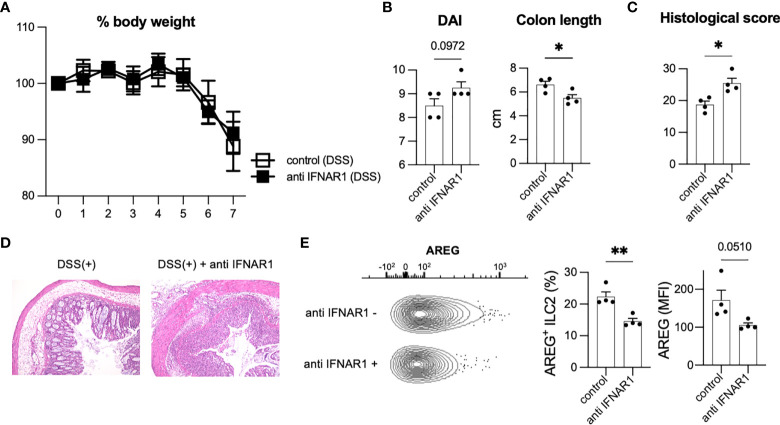
Inhibition of T1IFN signaling exacerbated DSS-induced colitis. WT mice were injected anti-IFN-alpha/beta receptor1 (IFNAR1) antibody intraperitoneally (i.p.) on day 0 and administered DSS (n=4 for each group). **(A)** Change in % body weight relative to initial body weight of control (DSS treated) mice (n=4) and anti IFNAR1 i.p. injected (DSS treated) mice (n=4). **(B)** Disease activity index (DAI) score on day 7 and colon length of mice of each group (n=4 for each group). **(C, D)** Histological score (n=4 for each group) and histopathology of distal colon. **(E)** Amphiregulin (AREG) expression in ILC2s of control mice and anti-IFNAR1 treated mice. Representative contour plots showing AREG expressing ILC2s in control mice (DSS treated) and anti IFNAR1 i.p. injected mice (DSS treated). Frequency of AREG expressing ILC2s among all ILC2s and mean fluorescence intensity (MFI) of AREG in ILC2s (n=4 for each group). Data are mean and SEM. *P < 0.05, **P < 0.01.

T1IFN acts as an anti-inflammatory immunomodulator with protective effects against colitis (23, 24). T1IFN may be associated with the phenotypic changes that occur in ILC2s to impart protection against colitis. To analyze the effects of T1IFN signaling on ILC2s during colitis, we blocked T1IFN signaling in mice by i.p. injecting anti-IFNAR1 antibody to mice on day 0 and administered DSS. Although no significant reduction in body weight was observed (**Figure 3A**), injection of anti-IFNAR1 antibody resulted in increased DAI and shortened colon length (**Figure 3B**) compared with that in control DSS mice. Histology also showed exacerbation of colitis in anti-IFNAR1 treated mice compared with that in control mice (**Figures 3C, D**). Consistent with WT mice (**Figures 3A–D**), neutralizing T1IFN signaling resulted in a more sever colitis histology in *Rag2*-deficient mice (**Supplementary Figures 3A–D**).

We next investigated the potential contribution of ILC2s in exacerbating colitis. Blocking of IFNAR1 reduced AREG-expressing ILC2s in WT and *Rag2*-deficient mice (**Figure 3E**, **Supplementary Figure 3E**), which have been reported to play critical roles in DSS-induced colitis (20). In addition, ILC2s are a major source of AREG in hematopoietic cells in the colon (**Figures 1D, E**). Eosinophils and mast cells produce AREG in some allergic models challenged by specific antigens; however, there are very few eosinophils and mast cells in the colon of healthy and colitis C57BL/6 WT mice (**Supplementary Figure 3F**). Next, we evaluated the colonic ILC2-derived AREG expression following type I IFN stimulation *in vitro* and observed that IFN stimulation significantly increased AREG expression in colonic ILC2s (**Supplementary Figure 3G**). We also reanalyzed publicly available RNA-seq data (GSE73272) (25) and found that IFN-γ, another STAT1-activating cytokine, induced *Areg* expression in ILC2s (**Supplementary Figure 3H**). These data suggest that T1IFN signaling is important for AREG production in colonic ILC2s and protection of the host from intestinal inflammation.

### Exogenous AREG rescued severity of DSS-induced colitis exacerbated by neutralization of T1IFN signaling

**Figure 4:**
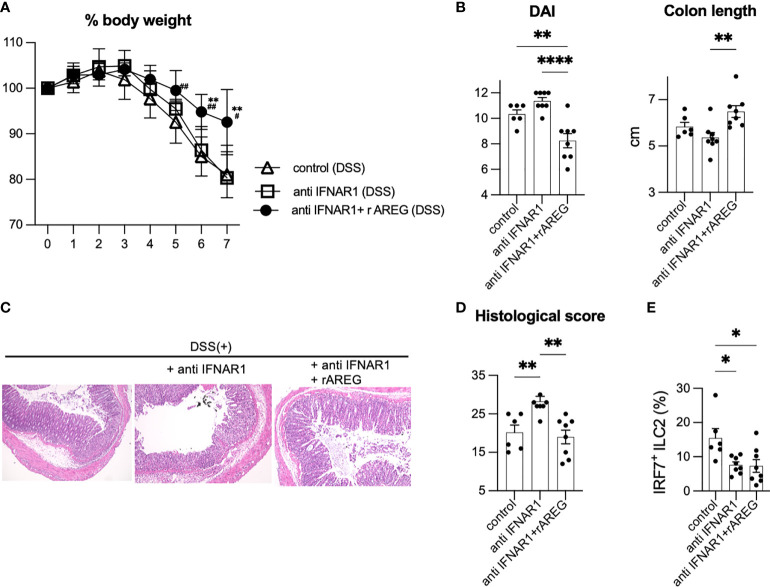
Amphiregulin treatment partially rescued severity of DSS-induced colitis in anti-IFNAR treated mice. WT mice were injected exogenous recombinant AREG (rAREG) over the course of DSS exposure with anti-IFNAR1 i.p. injection. Data are representative of two independent experiments (n=6 for control group, n=8 for anti IFNAR1 only and anti IFNAR1 plus rAREG injected group). **(A)** Change in % body weight relative to initial body weight of control mice (DSS treated) (n=6), anti IFNAR1 i.p. injected mice (DSS treated) (n=8) and anti IFNAR1 plus rAREG i.p. injected mice (DSS treated) (n=8). ^**^ *P*<0.01 vs anti IFNAR1 group, ^#^ *P*<0.05, ^##^ *P*<0.01 vs control group. **(B)** Disease activity index (DAI) score on day 7 and colon length of mice of each group. **(C, D)** Histological score and histopathology of distal colon. **(E)** Frequency of IRF7^+^ ILC2s in each group. Data are mean and SEM. *P < 0.05, **P < 0.01, ****P < 0.0001.

To confirm the protective role of AREG in intestinal inflammation by neutralizing T1IFN signaling, WT mice were treated with exogenous recombinant AREG (rAREG) over the course of DSS exposure. Administration of rAREG significantly ameliorated body weight loss (**Figure 4A**) and DAI (**Figure 4B**) in the anti-IFNAR1 treated DSS mice. Shortened colon length in anti-IFNAR1 treated DSS mice was diminished in rAREG-treated DSS mice (**Figure 4B**). Histological scoring confirmed the improvement owing to rAREG treatment (**Figures 4C, D**). Unlike lung ILC2s in allergic inflammations (26), IRF7 expressing ILC2s in the colon were reduced in anti-IFNAR1 treated mice compared to that of control mice, regardless of rAREG treatment (**Figure 4E**, **Supplementary Figure 4**), which indicates that exogenous rAREG does not recover the downregulation of T1IFN signaling in ILC2s. Collectively, these results demonstrate that the T1IFN signaling exacerbation of colitis was partly due to AREG in an ILC2-dependent manner.

## Discussion

The roles of ILC2s in the intestinal tract have been extensively studied in the context of helminth infections (7, 18, 27). These studies have revealed the critical roles of ILC2s during type 2 immune responses and mainly focused on type 2 cytokines, such as IL-5 and IL-13, which induce eosinophilia and goblet cell hyperplasia. However, the roles of ILC2s in intestinal inflammation, except for type 2 inflammation, remains unclear. Herein, we revealed a previously unrecognized role of ILC2s in controlling the pathogenesis of acute intestinal inflammation. ILC2s are the most abundant cells in the colon among visceral organs, and ILC2s obtained from the colonic mucosa under inflammatory conditions showed significant enrichment of T1IFN signaling. Neutralization of T1IFN signaling exacerbated colitis severity accompanied by a reduction in AREG-producing ILC2s in DSS-induced colitis, which was ameliorated by exogenous treatment with AREG.

ILC2s play a central role in the formation of type 2 immune responses during infections by parasites and fungi and allergic conditions in the lungs and other organs. Multiple factors contribute to the activation and repression of ILC2s. ILC2s proliferate and produce IL-5 and IL-13 upon IL-25 and IL-33 stimulation, which are suppressed by STAT1-dependent cytokines such as T1IFN, IFN-γ, and IL-27 (25, 28). Transcriptomic analysis unbiasedly revealed the enrichment of genes related to T1IFN signaling in ILC2s during colitis. Our data suggests that ILC2s show enhanced production of genes related to T1IFN responses and a reduction in IL-5 production in colitis. Our findings indicate that upregulation of T1IFN alters the ILC2 phenotype.

AREG plays a critical role in wound repair and tissue remodeling by promoting epithelial cell proliferation (29). Despite the importance of AREG production by hematopoietic cells in the context of mucosal damage, regulation of AREG expression in ILC2s is not yet fully understood, and the roles of STAT1 dependent cytokines, including IFN-β, IFN-γ, and IL-27, show inconclusive results for AREG production in ILC2s (25, 28). A recent report suggested that long-term stimulation with IFN-β enhanced AREG production (30). Consistently, GO analysis showed enrichment of genes related to T1IFN signaling and a decrease in AREG expression in ILC2s upon neutralization of T1IFN signaling, providing an additional aspect of the protective roles of ILC2s through T1IFN signaling *in vivo*. Consistently, adoptive transfer of ILC2s expanded in mice treated with IL-33 into mice with DSS-induced colitis, resulting in a significant improvement in the severity of colitis compared to mice without ILC2 transfer (31). Although both pro- and anti-inflammatory roles of T1IFN in intestinal inflammation have been reported (32), and clinical trials have shown inconclusive results for T1IFN in IBD patients (33), tissue- or cell type- specific manipulation of T1IFN signaling or AREG supplementation might be a potential therapeutic option for treating IBD or other intestinal inflammatory diseases.

ILC2s have been reported to exacerbate colitis (34, 35) and promote wound healing (20, 36). Recent reports suggest that cytokines known to activate ILC2s, such as IL-33 and thymic stromal lymphopoietin (TSLP), have protective effects against DSS-induced colitis (20, 37). It was unexpected that T1IFN, known to repress type 2 cytokine production in ILC2s, enhances AREG production in ILC2s *in vivo*. AREG is expressed by intestinal epithelial cells (29); however, type III IFN, rather than type I IFN, induces the nuclear translocation of STAT1 in colonic epithelial cells (38). Therefore, it is possible that T1IFN and AREG act on both colonic epithelial cells and hematopoietic cells. In addition to epithelial cells and ILC2s, there are several hematopoietic cell types that are known to produce AREG, such as eosinophils, mast cells, and T cells (39–42). However, we detected a minimal number of eosinophils and mast cells in colitic *Rag2*-deficient mice, in which blocking T1IFN worsened colitis. In addition, scRNA-seq analysis showed that ILC2s are a major source of AREG among hematopoietic cells, suggesting that ILC2s are a major producer of AREG upon T1IFN signaling in hematopoietic cell populations in the colon. A limitation of this study is the lack of evidence regarding the importance of ILC2-derived AREG in colitis. Although the generation of ILC2-specific deletions of AREG is technically challenging, future studies are expected to reveal an ILC2-specific role of AREG in intestinal homeostasis. Another issue that remains to be clarified is that although colonic ILC2s produce AREG upon T1IFN stimulation *in vitro*, colitis itself does not induce AREG. We speculate that AREG production is induced or repressed by multifactorial cytokine networks during colitis; however, leastwise, “tonic T1IFN signaling” (43, 44) is important in maintaining AREG production; thus, blocking T1IFN signaling significantly reduced AREG production in colonic ILC2s.

In summary, this study is the first to demonstrate the anticolitic role of T1IFN signaling in ILC2s during acute intestinal damage. AREG production mediates the anticolitic phenotype of ILC2s. However, the effect of the neutralization of T1IFN signaling in the chronic phase has not been clarified because the functions of ILC2s are different in the acute and chronic phases of inflammation (45). The role of T1IFN in colonic ILC2s found in a murine colitis model is expected to be further explored in human diseases.

## Data availability statement

RNA sequencing data have been deposited to Gene Expression Omnibus under the accession number GSE212939.

## Ethics statement

The animal study was reviewed and approved by Animal study committees (Keio University).

## Author contributions

EI and YoM designed the experiments and interpreted data. EI, RI, SH, IM, YH, YT, JT, KI, YuM, YY, HK, TS, and TT performed the experiments. EI, IM, and YoM analyzed the genomic data and generated the figures. KT, NH, HO, NN, and TK supervised and supported this study. EI drafted the manuscript. YoM wrote the manuscript with input from other authors. All authors contributed to the article and approved the submitted version.

## Funding

This study was funded by the Japan Society for the Promotion of Science (JSPS) KAKENHI (B) 20H03666 to YoM, and (A) 20H00536 to TK; JSPS Grant-in-Aid for Transformative Research Areas(B): 21H05123 to YoM.; Advanced Research and Development Programs for Medical Innovation (AMED-CREST: 21gm1510002h0001to TK, and 20gm1210001h0001 to YM; the Practical Research Project for Rare/Intractable Disease: 21ek0109556h0001 to YM); the Japan Foundation for Applied Enzymology; and Keio University Medical Fund.

## Acknowledgments

We thank R. Sakakibara, S Suzuki, Y. Kaieda, H. Suzuki, K. Ono, S. Tanemoto, M. Ichikawa, K. Miyamoto, and Y. Harada (Keio University) for technical assistance. We thank C. Ido and the animal facility staff (Keio University) for their technical support in handling mice. We thank Kazuyo Moro (Osaka University), Giuseppe Sciumè (Sapienza University of Rome), Hiroki Kabata (Keio University), and the members of the Kanai laboratory for their helpful suggestions. We would like to thank Editage (www.editage.com) for English language editing.

## Conflict of interest

The authors declare that the research was conducted in the absence of any commercial or financial relationships that could be construed as a potential conflict of interest.

## Publisher’s note

All claims expressed in this article are solely those of the authors and do not necessarily represent those of their affiliated organizations, or those of the publisher, the editors and the reviewers. Any product that may be evaluated in this article, or claim that may be made by its manufacturer, is not guaranteed or endorsed by the publisher.

## Supplementary material

Supplementary Figure 1
**(A)** Frequency of Lin^–^CD127^+^ total ILCs among CD45^+^ lymphocytes in the colon (LI), small intestine (SI), liver, spleen, and MLN of SPF mice. n=5 for each tissue. Data are representative of two independent experiments(mean and SEM). ****P < 0.0001. **(B)** UMAP (Uniform ManifoldApproximation and Projection) of representative gene expression for T cells *(Cd3e)*, B cells *(Cd19)*, plasma cells *(Igha)*, macrophages and dendritic cells *(Csf1r, Itgam, Itgae)*, T cells and ILCs *(Il7r)*, and ILC2s *(Rora, Il5, Il13,Calca)*. (n=5 SPF mice).

Supplementary Figure 2
**(A)** Representative flow cytometry plots of cytokine production by ILC2s in the control and DSS-treated groups stimulated with PMA plus ionomycin. **(B)** Frequency of IL-5 and IL-13 producing colonic ILC2s in each group among all ILC2s. Graphs show pooled data from three independent experiments (n=12 for each group). **(C)** Frequencies of IL-5 producing colonic ILC2s among all ILC2s of each group stimulated with PMA plus ionomycin, IL-25, IL-33, and IL-25 plus IL-33 for 4h (n=4 for each group). **(D)** Gating strategy for the enrichment of colonic ILC2s. **(E)** Heatmap showing the relative expression of signature genes of ILC2s (22) and the number of upregulated (12 genes) and downregulated genes (20 genes) in DSS-treated mice compared to that in control mice (n=3 for each group). **(F)** TPM values of signature genes of ILC2s and other groups of ILCs. *P<0.05, **P<0.01, ***P<0.001.

Supplementary Figure 3 *Rag2*-deficient mice were injected anti IFNAR1 antibody i.p. on day 0 and administered DSS (n=4 for control group and n=5 for anti IFNAR1 i.p. injected group). **(A)** Change in % body weight relative to initial body weight of control mice (DSS treated) (n=4) and anti IFNAR1 i.p. injected mice (DSS treated) (n=5). **(B)** Disease activity index (DAI) score on day 7 and colon length of the mice in each group. **(C, D)** Histological score and histopathology of the distal colon. **(E)** Frequency of AREG expressing ILC2s among all ILC2s in each group. **(F)** The number of colonic ILC2s, eosinophil, and mast cells in control and DSS-treated *Rag2*-deficient mice (n=3 for each group). **(G)** Frequency of AREG expressing ILC2s among all ILC2s following 4h *in vitro* stimulation with or without IFNβ. 12 mice were used and 2 mice were pooled in one sample. n=3 for each group. **(H) **FPKM (fragments per kilobase million) values of *Il5, Areg, and Stat2* of ILC2s treated with IL-33 or IL-33 plus IFN-γ. Data are mean and SEM.

Supplementary Figure 4
**(A)** Representative contour plots showing IRF7 expressing ILC2s in control mice (DSS treated), anti IFNAR1 i.p. injected mice (DSS treated), and anti IFNAR1 plus rAREG i.p. injected mice (DSS treated). **(B)** MFI of IRF7 in ILC2s (n=4 per group from one experiment). **(C)** Absolute number of IRF7^+^ ILC2s in each group. Graphs show pooled data from two independent experiments (n=6 for control mice, n=8 for anti IFNAR1 i.p. injected mice and anti IFNAR1 plus rAREG i.p. injected mice). Data are mean and SEM.
