# Supplementary material for: Enrichment of type I interferon signaling in colonic group 2 innate lymphoid cells in experimental colitis
Source: Front Immunol. 2022 Oct 4;13:982827. doi: 10.3389/fimmu.2022.982827 (PMC9578145; doi:10.3389/fimmu.2022.982827)
Supplement: Supplementary file 1 [file Table_1.pdf]

Supplementary table 1. List of antibodies used in this study

	antibody	Clonality	clone number	conjugate	vendor	catalog number
CD3	anti- CD3e antibody	monoclonal	145-2C11	BV510	BD Biosciences	563024
CD3	anti- CD3e antibody	monoclonal	145-2C11	BV785	Biolegend	100355
B220	anti- B220 antibody	monoclonal	RA3-6B2	APC-Cy7	BD Biosciences	552094
CD19	anti- CD19 antibody	monoclonal	1D3	APC-Cy7	BD Biosciences	557655
CD45	anti- CD45 antibody	monoclonal	30-F11	BV650	BD Biosciences	103151
CD5	anti- CD5 antibody	monoclonal	53-7.3	BV510	BioLegend	100627
CD4	anti- CD4 antibody	monoclonal	RM4-5	FITC	BD Biosciences	553047
CD127	anti- CD127 antibody	monoclonal	SB/199	BV421	BD Biosciences	562959
NK1.1	anti- NK-1.1 antibody	monoclonal	PK136	PE	eBioscience	12-5941-82
KLRG1	anti- KLRG1 antibody	monoclonal	2F1	PE-Cyanine7	eBioscience	25-5893-80
NKp46	D335 (NKp46) a	monoclonal	29A1.4	PE	eBioscience	11-3351-80
ST2	anti- ST2 antibody	monoclonal	RMST2-2	PerCP	eBioscience	46-9335-82
IL-17RB	anti- IL-17RB antibody	monoclonal	9B10	PE	BioLegend	146315
IRF7	anti- IRF7 antibody	monoclonal	MNGPKL	PE	eBioscience	12-5829-82
Amphiregulin	anti- Amphiregulin antibody	monoclonal	206220		R &D	MAB989-100

Antibody Labelling Kit				Alexa Fluor 647	rmo Fisher Scier	A20186
Gata3	anti- Gata-3 antibody	monoclonal	L50-823	Alexa Fluor 488	BD Biosciences	560163
Gata3	anti- Gata-3 antibody	monoclonal	L50-823	BV421	BD Biosciences	563349
Rorγt	anti- RORγt antibody	monoclonal	Q31-378	BV421	BD Biosciences	562894
Foxp3	anti- FOXP3 antibody	monoclonal	FJK-16s	PerCP-Cyanine5.5	eBioscience	45-5773-82
IL-5	anti- IL-5 antibody	monoclonal	TRFK5	PE	BD Biosciences	554395
IL-13	anti- IL-13 antibody	monoclonal	eBio13A	PerCP-eFluor710	eBioscience	46-7133-82
IL-4	anti- IL-4 antibody	monoclonal	11B11	BV421	BioLegend	504119
IL-17A	anti- IL-17A antibody	monoclonal	TC11-18H10.1	BV510	BioLegend	506933
IL-22	anti- IL-22 antibody	monoclonal	IL22JOP	APC	eBioscience	17-7222-82
IFNγ	anti- IFN-γ antibody	monoclonal	XMG1.2	PE-Cy7	BD Biosciences	557649
Mouse BD Fc Block	anti- CD16/CD32 antibody	monoclonal	2.4G2		BD Biosciences	553142
	Live-or-Dye™ Fixable Viability (750/777)				Biotium	32008
