# Supplementary figures and images for: Enrichment of type I interferon signaling in colonic group 2 innate lymphoid cells in experimental colitis
Source: Front Immunol. 2022 Oct 4;13:982827. doi: 10.3389/fimmu.2022.982827 (PMC9578145; doi:10.3389/fimmu.2022.982827)

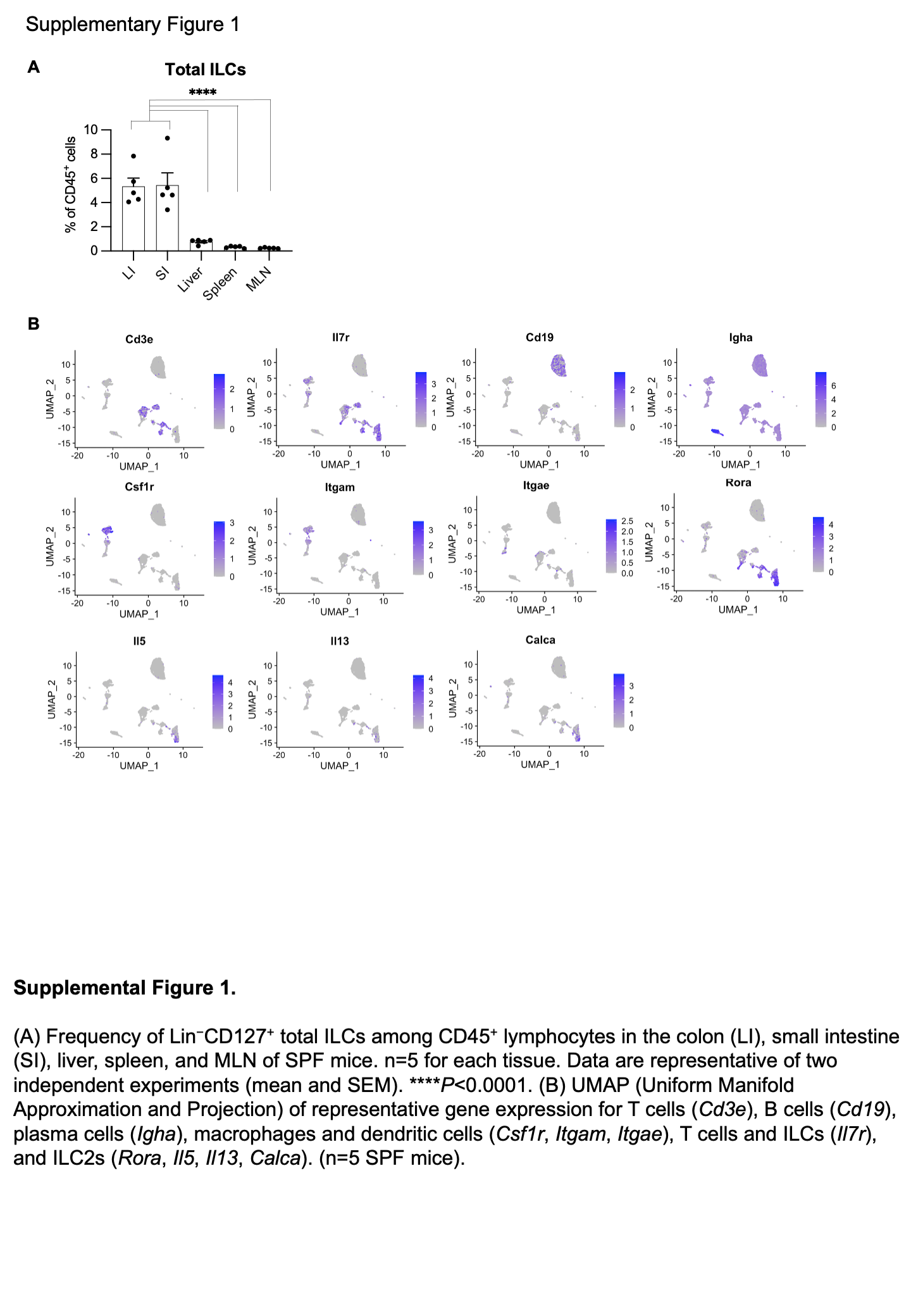

Supplement: Supplementary Figure 1 — (A) Frequency of Lin–CD127+ total ILCs among CD45+ lymphocytes in the colon (LI), small intestine (SI), liver, spleen, and MLN of SPF mice. n=5 for each tissue. Data are representative of two independent experiments(mean and SEM). ****P < 0.0001. (B) UMAP (Uniform ManifoldApproximation and Projection) of representative gene expression for T cells (Cd3e), B cells (Cd19), plasma cells (Igha), macrophages and dendritic cells (Csf1r, Itgam, Itgae), T cells and ILCs (Il7r), and ILC2s (Rora, Il5, Il13,Calca). (n=5 SPF mice). [file Image_1.tiff]

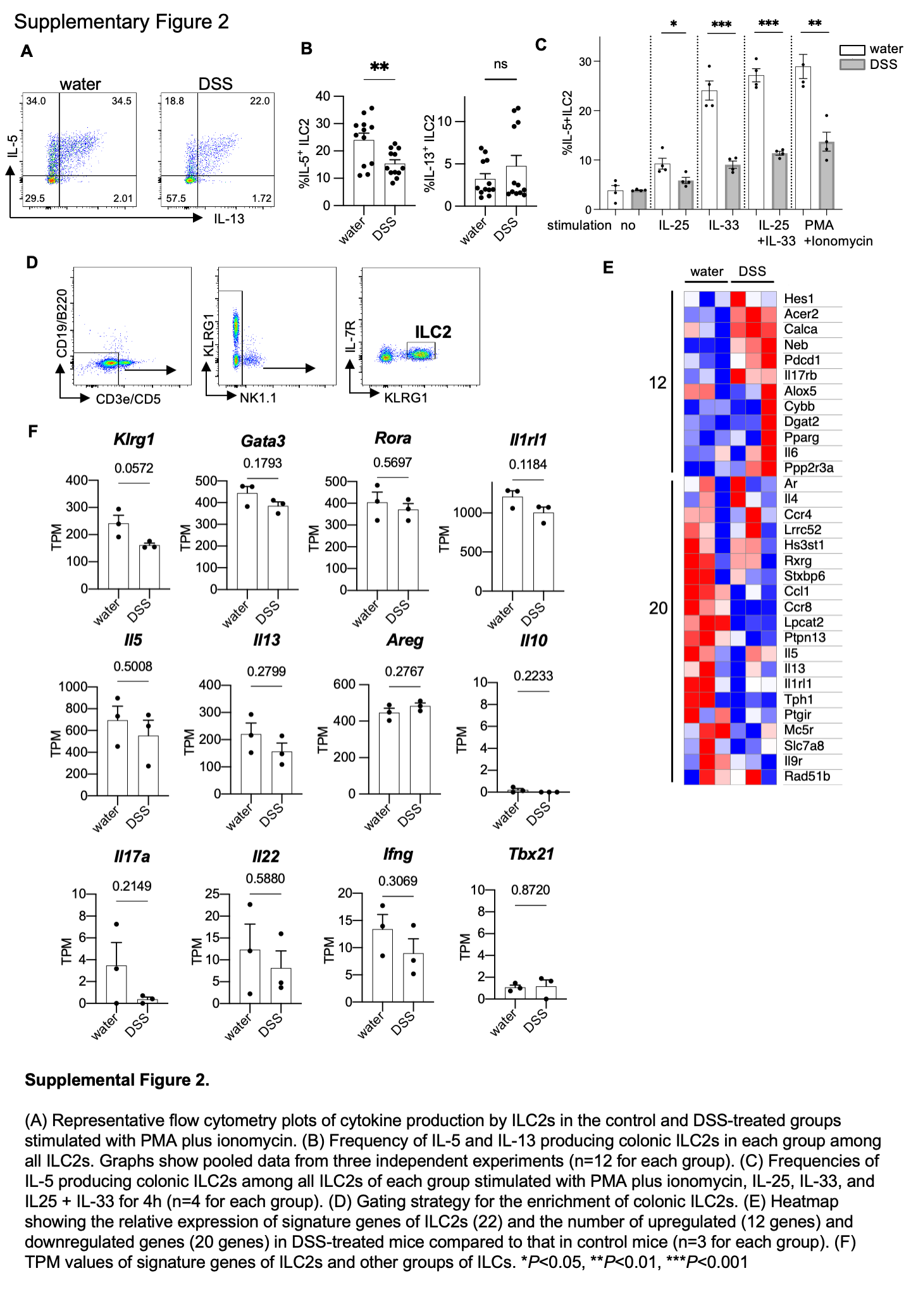

Supplement: Supplementary Figure 2 — (A) Representative flow cytometry plots of cytokine production by ILC2s in the control and DSS-treated groups stimulated with PMA plus ionomycin. (B) Frequency of IL-5 and IL-13 producing colonic ILC2s in each group among all ILC2s. Graphs show pooled data from three independent experiments (n=12 for each group). (C) Frequencies of IL-5 producing colonic ILC2s among all ILC2s of each group stimulated with PMA plus ionomycin, IL-25, IL-33, and IL-25 plus IL-33 for 4h (n=4 for each group). (D) Gating strategy for the enrichment of colonic ILC2s. (E) Heatmap showing the relative expression of signature genes of ILC2s (22) and the number of upregulated (12 genes) and downregulated genes (20 genes) in DSS-treated mice compared to that in control mice (n=3 for each group). (F) TPM values of signature genes of ILC2s and other groups of ILCs. *P<0.05, **P<0.01, ***P<0.001. [file Image_2.tiff]

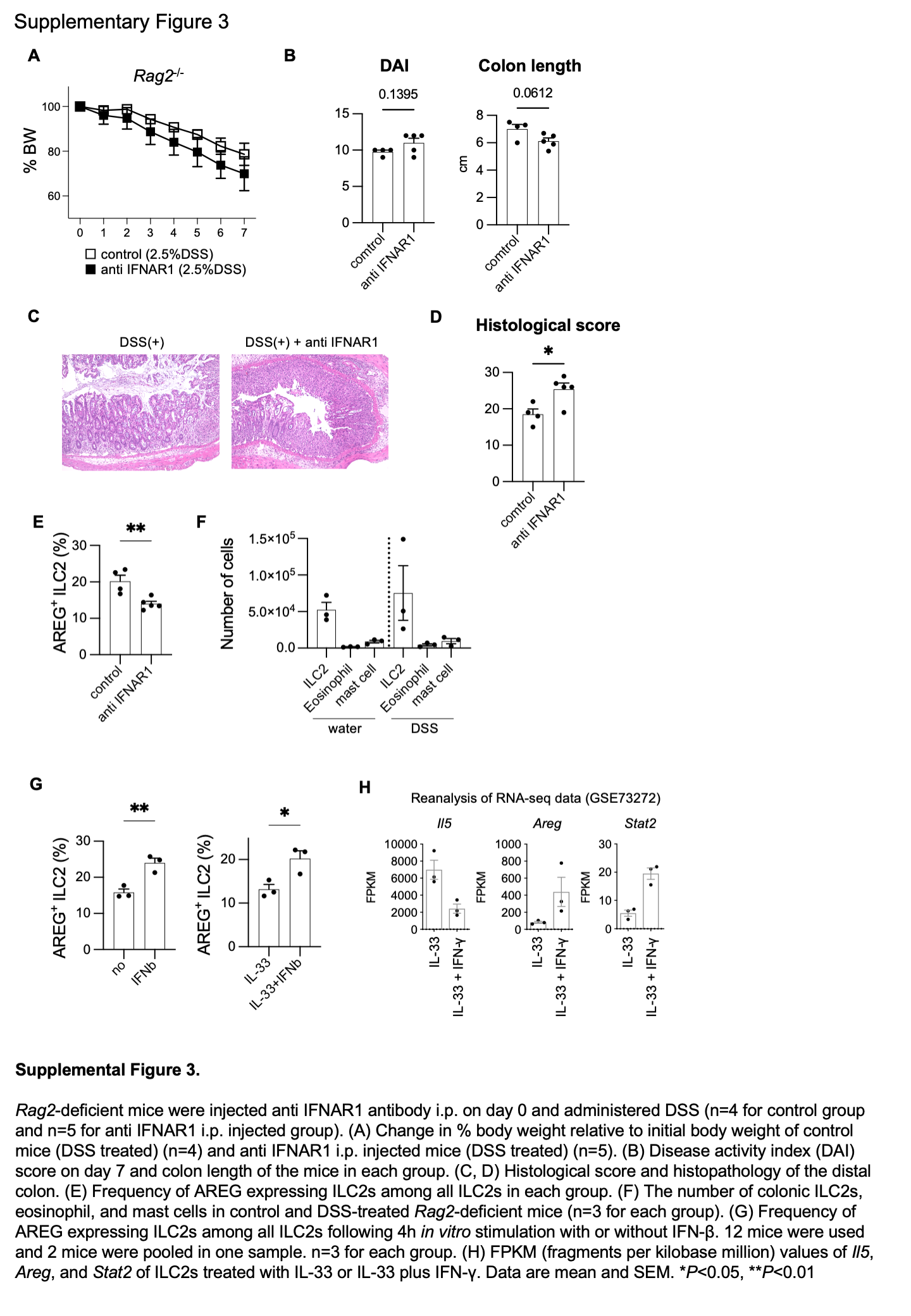

Supplement: Supplementary Figure 3 — Rag2-deficient mice were injected anti IFNAR1 antibody i.p. on day 0 and administered DSS (n=4 for control group and n=5 for anti IFNAR1 i.p. injected group). (A) Change in % body weight relative to initial body weight of control mice (DSS treated) (n=4) and anti IFNAR1 i.p. injected mice (DSS treated) (n=5). (B) Disease activity index (DAI) score on day 7 and colon length of the mice in each group. (C, D) Histological score and histopathology of the distal colon. (E) Frequency of AREG expressing ILC2s among all ILC2s in each group. (F) The number of colonic ILC2s, eosinophil, and mast cells in control and DSS-treated Rag2-deficient mice (n=3 for each group). (G) Frequency of AREG expressing ILC2s among all ILC2s following 4h in vitro stimulation with or without IFNβ. 12 mice were used and 2 mice were pooled in one sample. n=3 for each group. (H) FPKM (fragments per kilobase million) values of Il5, Areg, and Stat2 of ILC2s treated with IL-33 or IL-33 plus IFN-γ. Data are mean and SEM. [file Image_3.tiff]

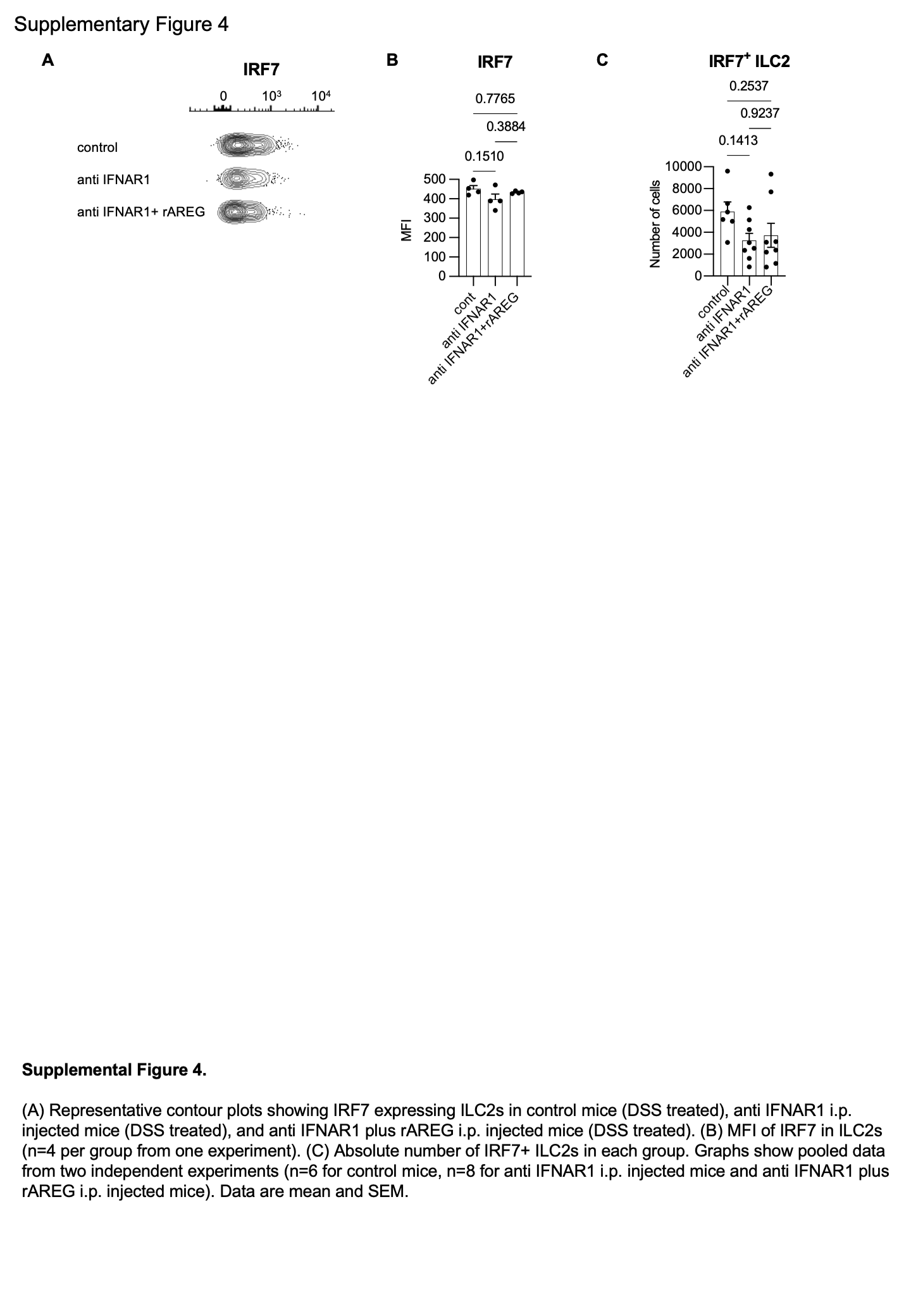

Supplement: Supplementary Figure 4 — (A) Representative contour plots showing IRF7 expressing ILC2s in control mice (DSS treated), anti IFNAR1 i.p. injected mice (DSS treated), and anti IFNAR1 plus rAREG i.p. injected mice (DSS treated). (B) MFI of IRF7 in ILC2s (n=4 per group from one experiment). (C) Absolute number of IRF7+ ILC2s in each group. Graphs show pooled data from two independent experiments (n=6 for control mice, n=8 for anti IFNAR1 i.p. injected mice and anti IFNAR1 plus rAREG i.p. injected mice). Data are mean and SEM. [file Image_4.tiff]
